# Control of Asthma and its Influencing Factors in Children Followed in Pneumo-pediatrics Consultation at the Mother-child Hospital in the City of Marrakech, Morocco

**DOI:** 10.2174/0118743064340759241209041049

**Published:** 2025-02-04

**Authors:** Maryem Labyad, Ghizlane Draiss, Karima El Fakiri, Nadia Ouzennou, Mohammed Bouskraoui

**Affiliations:** 1 Infectious Disease Research Laboratory, Faculty of Medicine and Pharmacy, Cadi Ayyad University, Marrakech, Morocco; 2 Pediatric Department, Faculty of Medicine and Pharmacy of Marrakech, University Hospital Mohamed VI, Cadi Ayyad University, Marrakech 40030, Morocco; 3 ISPITS, Higher Institute of Nursing and Technical Health, Marrakech, Morocco; 4 Department of Biology, Faculty of Sciences Semlalia, Pharmacology, Neurobiology, Anthropobiology and Environment Laboratory, Cadi Ayyad University, Marrakech, Morocco

**Keywords:** Asthma, Assessment, Child, Consultation, Morroco, trigger, influencing factors

## Abstract

**Introduction:**

Evaluate asthma control and determine its influencing factors to ensure adequate management and improve the quality of life for asthmatic children.

**Method:**

A prospective cross-sectional study was conducted over a two-month period, from 02/11/2022 to 01/01/2023, at the pneumo-pediatric consultation at the MCH. The target population was asthmatic children aged 4 to 11 and their parents. The Arabic version of the C-ACT and PMAQ-3W were used to assess asthma control and medication compliance, respectively.

Statistical analysis was performed with SPSS, using descriptive and correlational analysis (bivariate and multivariate).

**Results:**

203 asthmatic children were included in the study, out of which 60.6% were male, with a mean age of 6 years. Asthma was uncontrolled in 53% of children. Factors associated with uncontrolled asthma were rural residence, low parental education, low monthly family income, lack of awareness of triggers, presence of conjunctivitis or allergic rhinitis, occurrence of respiratory infections, parental history of asthma, exposure to smoking, and non-compliance with treatment.

**Conclusion:**

53% of children had uncontrolled asthma. A range of predictive factors were significantly associated with suboptimal asthma control. Recommended actions to improve childhood asthma control include education on trigger prevention and medication compliance, treatment of comorbidities, and accessibility of care for all socio-economic classes.

## 1 INTRODUCTION

Asthma is a heterogeneous disease characterized by chronic airway inflammation, with respiratory symptoms such as dyspnea, sibilant rales, bronchial hyperproduction, cough, and variable expiratory flow limitation [1]. Its prevalence is increasing significantly, especially in developing countries [2]. In Morocco, the AIRMAG study showed that the prevalence of asthma in children was around 4.4% [3]. A survey carried out in Morocco's 16 administrative regions from 2004 to 2012 reported that the average annual growth rate in asthma consultations among children in the city of Marrakech was 10% [4]. In the same city, another study showed that paediatric asthma accounted for 57.5% (92 cases) of allergy investigations between December 2002 and December 2005 [5].

Despite significant advancements in understanding asthma, its management in children remains inadequate [6]. Moreover, to address this, maintaining an optimal level of control [7] is the most recommended approach, as it correlates with an improved quality of life for children [8].GINA (Global Initiative of Asthma) has defined asthma control as the perceptible intensity of the effects of asthma on the patient or the degree of elimination of these effects using treatment. Additionally, it is based on daytime and nocturnal symptoms, the use of rescue medication, activity limitation, lung function, and the number of exacerbations [1].

Control of childhood asthma was not always maintained; the ER'ASTHME study reported that asthma control was unacceptable in 66% of cases [9]. In the African context, uncontrolled asthma was approved in 30.9%, 31%, and 44.3% of children in Nigeria, Ethiopia, and South Africa, respectively [10]. In Morocco, 61% of children in the city of Casablanca did not have well-controlled asthma [11]. However, no such study has been carried out in the city of Marrakech.

Patients with uncontrolled asthma are at greater risk of exacerbation, emergency room visits, and hospitalization [12]. In these patients, factors associated with loss of asthma control should be investigated [9], such as obesity, rhinitis, conjunctivitis, gastro-oesophageal reflux disease (GERD), food allergies, socio-economic status, and medication compliance [1]. The latter is one of the most common causes of uncontrolled asthma [12], as noted in several studies [13, 14].

## 2 OBJECTIVES

The aim of the present survey is to assess the level of asthma control and to explore its influencing factors in asthmatic children aged 4 to 11 years followed up in a pneumo-paediatric consultation at the Mother and Child Hospital (MCH) Marrakech.

## 3 MATERIALS AND METHODS

This is a prospective cross-sectional survey conducted over two months, from 01/11/2022 to 02/01/2023, at the pneumo-paediatric consultation at the MCH, part of the Marrakech University Hospital.

### 3.1 The Target Population

The study population was selected according to the following criteria:

Inclusion criteria:

- Children aged 4 to 11 years

- Children with a diagnosis of asthma at least 6 months prior to the start of the study to ensure adequate time to assess asthma control

- Children with an appointment during the month of data collection

- Children whose parents agreed to participate in the study

Exclusion criteria:

- Children with other respiratory problems

- Presence of cognitive or mental disorders that may influence the ability to understand questionnaire and C-ACT instructions.

### 3.2 Study Variables

The dependent variable was asthma control; patients were classified into 2 groups: controlled asthma and uncontrolled asthma. The independent variables included socio-demographic characteristics, which were classified based on Morocco's social indicators, elaborated by the Haut commissariat au plan (HCP), these variables encompassed: the child's age, gender, number of people in the household, place of residence (urban or rural), type of housing (villa, apartment, room, detached house), parents' level of education (illiterate, primary, secondary, university), and monthly income (< 2000 MAD, 2000 MAD <I<5000 MAD, I> 5000 MAD) [15].

Secondly, the clinical characteristics included age of asthma, family history of asthma, asthma-related emergency room visits, frequency of consultations (for this last variable, the appointment history was recorded using the computerized information system adopted at the University Hospital), asthma treatment, therapeutic education (TE) and compliance.

Thirdly, asthmatic children's exposure to triggers (passive smoking, presence of pets, respiratory tract infections and physical exercise) and comorbidities (rhinitis, allergic conjunctivitis, gastro-oesophageal reflux disease (GORD), food allergy, eczema) were included. The modalities of these two variables were determined on the basis of the literature [1, 16, 17].

### 3.3 Data Collection

In the present study, three tools were used to collect data:

- A structured questionnaire containing the following sections: sociodemographic characteristics, clinical characteristics, and asthmatic children's exposure to triggers and comorbidities.

- The Patient Medication Adherence Questionnaire over the last 3 days and the preceding weekend (PMAQ-3W) is a validated tool for assessing medication adherence [18]. It consists of five questions with three response categories: total compliance if all doses were taken, partial compliance if one dose was missed, and non-compliance if more than one dose was missed [19].

- Childhood Asthma Control Test (C-ACT) is a validated tool for assessing asthma control in a paediatric population aged 4 to 12 years [20]. and especially for identifying children whose asthma is poorly controlled; its specificity and sensitivity rates are 74% and 68%, respectively. The test consists of seven questions, four for children and three for parents, and is based on a score ranging from zero to 27; a score of less than 20 indicates uncontrolled asthma [21]. The Arabic version of C-ACT was used in the present study. This version has already been validated in the United Arab Emirates (UAE) and presents good reliability and validity using a single optimal threshold of 20 [22]. The use of the Arabic version in the present study was preceded by a cultural adaptation. The test items were retained, except for the addition of asthma signs in question 5, intended for parents, which were not present on the screen. The version obtained was then tested with 20 children and their parents to ensure that all the test items were correctly understood and acceptable.

In the waiting room, the Arabic-language C-ACT was filled in by each child and the parent present, and then each patient communicated his or her completed test to his or her attending physician at the time of consultation. At this point, the doctor and interviewer completed the structured questionnaire and the PMAQ-3W questionnaire.

### 3.4 Statistical Analysis

This was carried out using Statistical Programme for Social Sciences (SPSS) for Windows version 22. Univariate analysis was used to describe and summarize the results of the study; continuous variables were described by mean, and standard deviation and categorical variables were defined by absolute and relative frequencies.

In order to investigate the existence of a relationship between the level of asthma control and the other variables, a bivariate analysis was used; the Students' correlation coefficient was adopted to study the correlation between the level of control and the quantitative variables. The Chi 2 test was used to search for correlations between qualitative variables.

A *p-*value < 0.05 was considered the threshold of statistical significance for the various tests used. Statistically significant variables were subjected to multivariate logistic regression analysis using the odds ratio (OR), with a 95% confidence interval (CI), to elucidate the strength of these associations.

## 4 RESULTS

During the data collection period, from November 1, 2022 to January 02, 2023, the MCH received 225 asthmatic children at the pneumo-pediatric consultation. Twenty-two asthmatic children were excluded from the study, of whom 18 (8%) were under 4 years of age, and four parents chose not to participate. Finally, 203 children with asthma were included in the study, meeting the established inclusion criteria.

### 4.1 Socio-demographic Characteristics

The majority of children included in the study (60.6%) were male. The mean age was 6 years, with a standard deviation of ±1.5. More than half the families (61%) lived in urban areas, and 76.4% of the children lived in apartments. Most fathers had primary education (36.4%), while the majority of mothers (44.4%) had secondary education Table **1**.

### 4.2 Comorbid Conditions and Asthma-related Triggers

Comorbidities were present in 71.4% of asthmatic children. The most prevalent comorbidity was allergic rhinitis (65%). Table **2** lists the other comorbidities.

Nearly 68% of parents were unaware of their children's asthma triggers. The most frequently reported triggers included upper respiratory tract infections (53%) and exposure to smoking (47.8%).

### 4.3 Consultations and Previous use of Emergency Care Settings Due to Asthma

Consultations were scheduled every three months in 64% of cases, while 35.5% of children had bimonthly appointments. In addition, more than half of asthmatic children (53%) visited the emergency department at least once during the past year. Of these, 43% visited the emergency department on two or more occasions, as shown in Table **2**.

### 4.4 Therapeutic Management and Compliance

The majority of children in the present study (94%) were taking inhaled corticosteroid-based background treatment, adapted in stages according to the latest GINA recommendations, while only 12 children were not prescribed background treatment. Furthermore, 38% of children declared total adherence to their background treatment, 18% had moderate adherence, and the majority of children (44%) had no adherence at all (Table **2**).

### 4.5 Family History of Asthma

62.6% of children had a family history of asthma, with parental history accounting for 39%. The proportions of other family members affected by asthma are detailed in Table **2**.

### 4.6 Children's Level of Asthma Control

**Fig. (1):**
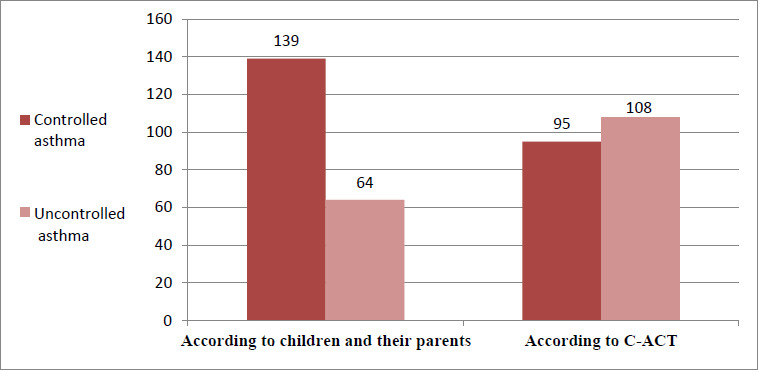
Asthma control according to C-ACT and the perception of children and parents.

More than half of asthmatic children (53%) had uncontrolled asthma based on the C-ACT score, while most children or their parents (68.5%) rated their asthma as “good” in response to the question “How is your asthma?” (Fig. **1**).

### 4.7 Factors Associated with Asthma Control in Children

A number of independent predictors of asthma control were revealed by multivariate analysis (Table **3**).

*Socio-demographic Characteristics:* Urban children were more likely to have controlled asthma than rural children (OR =0.51; 95% CI; 0.286 - 0.90; P = 0.022). In addition, low monthly family income put asthmatic children three times more at risk of uncontrolled asthma (OR =3.030, 95% CI; 1.035 - 8.870; P = 0.043). Low parental education was also associated with poor asthma control, according to the results reported in Table **3**.

*Clinical characteristics:* The existence of a family history of asthma was a risk factor for the child to have uncontrolled asthma (OR = 1.879; 95% CI; 1.057 - 3.341; P =0.032), this history was more specifically parental, either from the mother (OR = 2.234; 95% CI; 1.004 - 4.972; P =0.049) or the father (OR = 2.259; 95% CI; 1.035 - 4.931; P = 0.041). Also, children who complied with treatment were more likely to have controlled asthma (OR = 0.018; 95% CI; 0.007 -0.047; P =0.000). Similarly, those who did not visit the emergency department were more likely to have controlled asthma (OR =0.003; 95% CI; 0.001 -.007; P =0.000).

Comorbid conditions and asthma-related triggers: Children with allergic rhinitis or conjunctivitis were more likely to have uncontrolled asthma (OR =88.148, 95% CI; 25.734-301.935; P = 0.000), (OR =4.190; 95% CI1.912 -9.185; P =0.000) respectively. In addition, children with isolated asthma without comorbidities were more protected against uncontrolled asthma than those with comorbidities (OR =0.013; 95% CI; 0.003- 0.056; P = 0.000). Furthermore, the absence of recurrent respiratory infections was considered a protective factor against uncontrolled asthma (OR =0.002; 95% CI; 0.000- 0.007; P = 0.000). The knowledge of triggers by children or their parents was also a protective factor (OR =0.022; 95% CI; 0.007- 0.064; *P* = 0.000).

## 5 DISCUSSION

The objectives of the present study were to assess the level of asthma control and to identify associated factors in asthmatic children aged 4 to 11 years who were present at the pneumo-pediatrics consultation of Marrakech University Hospital at the time of the study.

Our results showed that more than half of the children had uncontrolled asthma and that factors such as rural residence, low level of education, low monthly parental income, lack of awareness of triggers, presence of co-morbidities, especially conjunctivitis and allergic rhinitis, the occurrence of respiratory infections, parental history of asthma, exposure to smoking and therapeutic non-compliance were significantly associated with suboptimal asthma control.

Data from our study revealed that asthma control in children was not maintained in 53% of the cases, in contrast to other studies conducted internationally. In a U.S. study of 160 children, a comparable rate of 51.88% of unmaintained control was reported. [23]. A higher rate of uncontrolled asthma (62.6%) was demonstrated in a survey of 278 asthmatic children in Saudi Arabia [24]. The AIRE study of 753 children in a European pediatric population also showed that uncontrolled asthma was the most prevalent, with a higher rate (66%) [25]. However, the results of our study were not similar to the results of a recent Ethiopian study of 105 asthmatic children, where only 31% had uncontrolled asthma [26]. Similarly, a survey in Nigeria showed a prevalence of suboptimal asthma control of 17.9% among 106 asthmatic children [27]. These variations could result from differences in environmental factors, treatment approaches, standards of medical care, or the specific characteristics of the populations studied.

In summary, the results of our study raise important questions about the variability of pediatric asthma control worldwide, highlighting the need for a deeper understanding of the underlying factors influencing the management of this disease in children.

The difficulty of maintaining an optimal level of asthma control may explain the high use of emergency departments by the children in our study (45% of children visited the emergency department more than 2 times) during the past year. This number ranged from 3.9 to 3.2 times a year in a Saudi study of 297 asthmatic children attending emergency departments, most of whom had uncontrolled asthma (60.3%) [28]. Likewise, a European study also highlighted a significant association between emergency room visits and uncontrolled asthma [29]. As demonstrated by a national study conducted in the city of Rabat, which included 1,461 asthmatic children, better asthma control can actually prevent the need to visit emergency rooms for asthma exacerbations [30].

The question “How is your asthma?” is deficient in terms of the quality and quantity of information it provides, both in its formulation and in the responses it elicits to adequately assess disease control. This can result in an overestimation of the level of asthma control, as shown in our survey and the ER'ASTHME study, where 38% of cases reported poor asthma control, while doctors estimated the rate to be 66% [31]. This can lead to an underestimation of the severity of the disease and to inadequate therapeutic management. Based on these data, it is therefore important not to restrict ourselves to general questions but rather to use scientifically validated tools to assess asthma symptoms and analyze its predictive factors in order to define personalized asthma control objectives [31, 32].

In fact, collaborative work between parents and healthcare professionals seems necessary to help parents (or children) better understand asthma symptoms, and maintain a better level of control. Additionally, ET is a type of collaboration between healthcare professionals and parents. It should be carried out at every consultation, as it enables skills to be acquired and maintained for optimal management of infantile asthma [33]. ET has a positive impact on children's asthma control and quality of life [34]. A Canadian study confirmed that asthma patients who participated in an ET program had lower rates of primary care and emergency room visits compared to those who did not [35]. Despite all these advantages, only 26.6% of parents reported benefiting from ET sessions. Several factors may be at the root of this result, such as logistical, economic, and educational barriers. These barriers were not explored in the present study and could be investigated in future research, highlighting the importance of a global approach to asthma management in children that incorporates ET within a broader context.

Our results demonstrated that the level of asthma control in children was linked to the parents' educational level, an essential factor previously recognized for maintaining effective asthma control in children [27]. A higher educational level facilitates understanding and adherence to the action plan provided by the doctor [36]. This underscores the necessity of providing comprehensive education for parents to enhance their understanding of the disease and ensure full adherence to its management.Similarly, low socioeconomic status is also among the predictors of suboptimal asthma control.. As supported by the present study and a study in Saudi Arabia, which indicated that household incomes below SAR 15,000 increase the risk of uncontrolled asthma by 2.30 times [37]. Similarly, in the U.S., parental unemployment and low income have been shown to contribute to poor asthma control [38]. In fact, low socio-economic status exposes caregivers to the risk of not being able to meet their financial obligations in terms of purchasing appropriate medications for optimal asthma management [39]. Indeed, making childhood asthma care accessible and affordable to all socio-economic classes in the community plays an essential role in ensuring optimal control of this condition.

In addition, our data showed a significant difference in asthma control between residential settings, with a higher rate of uncontrolled asthma in rural areas. These findings concur with those of a comparative study of asthma control in these two environments, adding that poor asthma control in rural areas was essentially due to difficulties in accessing care [40]. An investigation of the possible factors behind the difference in control between the two living environments will be necessary, which may concern geographical distance from health services, lack of adequate medical infrastructure, or other socio-economic barriers. This highlights the need for public health policies focused on reducing geographical disparities in pediatric asthma management, ensuring a better quality of life for all children, regardless of their location.Regarding therapeutic management, the majority of asthmatic children in our study were treated with inhaled corticosteroids, as this medication is the cornerstone of background treatment for childhood asthma [32]. In a study aimed at determining physicians' asthma treatment practices, 68% of patients were treated with this method [41], and 62% in another study of 153 asthmatic children [42]. Furthermore, inhaled corticosteroids offer many benefits for children with asthma, reducing the frequency and severity of asthma symptoms, as well as the risks of exacerbation and death caused by asthma [1]. However, despite all these advantages, our results revealed that only 38% of children complied with their treatment, which negatively influenced the maintenance of good asthma control. This observation is already documented, as therapeutic compliance is often insufficient, with adherence rates around 50%. [43]. Likewise, the ER’ASTHME study confirmed that only 57% of patients declared complete compliance with their treatment [9]. Similar to our investigation, several studies also documented poor medication adherence as a risk factor for uncontrolled asthma in children [28, 44, 45]. Consequently, the assessment of therapeutic compliance must be conducted systematically during consultations, particularly when asthma remains uncontrolled despite what is considered optimal therapeutic management [44, 46]. This was demonstrated in our study, where 54% of children had uncontrolled asthma despite being prescribed basic treatment.It is, therefore, necessary to develop and implement preventive strategies in order to improve therapeutic compliance in asthmatic children. An example of these strategies is the system that was implemented in a pulmonology department in Belgium, which aims to educate patients and their families while facilitating self-assessment of asthma control and rapid prescription of adaptive treatment plans for various asthma situations [43]. Likewise, electronic monitoring devices (EMDs) will likely be used more frequently to remind patients to take their medications as a strategy to motivate patients to maintain better medication adherence [44].Several reasons for non-compliance with therapy have already been highlighted, such as forgeting controller medications and not knowing prescribed doses [26]. However, our study did not explore these aspects; their investigation could be considered in future research to better understand their role in the insufficient control of asthma in children. 

Among the conclusions drawn from our study, we identified the negative impact of the presence of asthma in one of the parents on asthma control. This finding has also been confirmed by previous studies [26, 47]. Parents with a history of asthma may be less concerned about their children's asthma symptoms due to their familiarity with these symptoms [48]. Furthermore, this finding could be linked to genetic and environmental factors that could be transmitted within the family. This involves the application of more individualized prevention and management approaches aimed at improving disease control in people with a family history of asthma.

Knowledge of triggering factors by both children and their parents is crucial for maintaining better asthma control [27]. This was confirmed by our study, which showed that asthma control was poor in children and parents who were unaware of the triggering factors. Indeed, the avoidance of these factors is an essential element in the management of childhood asthma in addition to medical treatment and TE [49]. Among the triggering factors that were highlighted in the present study were recurring respiratory infections and exposure to tobacco smoke.

Many studies have reported that respiratory infections cause an exaggeration of asthma symptoms by leading to exacerbations and negatively affect asthma control [48, 50]. Similarly, a study of 408 children from secondary care centers in the Netherlands showed that viral respiratory tract infections were detected in association with 85% of asthma exacerbations [51]. Also, in another case-control study involving 133 children, 61% of children hospitalized for asthma attacks tested positive for rhinovirus [52]. It is therefore important to take measures to prevent respiratory infections in children with asthma, such as flu vaccination and following hygiene measures, and to closely monitor asthma symptoms during periods of respiratory infections [53].

Similar to our results, several previous investigations have claimed that exposure of asthmatic children to tobacco smoke had a negative impact on their asthma control [54, 55]. This impact was confirmed by a survey of 282 asthmatic children, which concluded that banning smoking in rooms used by asthmatic children is a simple way to reduce asthma symptoms and the use of anti-asthma medications [56]. In the same sense, and after the implementation of English anti-smoking legislation, a study approved a reduction in the number of admissions for childhood asthma of 6,802 hospitalizations during the first three years following the implementation of this legislation [57]. Moreoverit is important to protect asthmatic children from exposure to tobacco smoke by avoiding smoking inside the home and limiting their exposure in other environments. Hence, the crucial role of the attending physician in educating and raising awareness among parents regarding this issue

Identification of asthma-related comorbidities is an essential part of the assessment of difficult-to-control asthma in children [58]. These conditions are often overlooked despite the challenges that can present them, including diagnostic confusion due to mimicking asthma symptoms [59]. In addition, substantial health costs can be five times the costs attributable to asthma alone [60]. In our study, among the comorbidities evaluated, two negatively affected children's asthma control, namely allergic rhinitis and conjunctivitis

Indeed, asthma and allergic rhinitis are often linked, which has led to the common use of the term “united respiratory tract disease” [61]. In addition, by worsening asthma symptoms, allergic rhinitis is significantly associated with uncontrolled asthma [62].

The literature indicated that children with asthma and rhinitis have more frequent emergency department visits, hospitalizations, and higher asthma-related medication expenditures [63]. Also, it has already been approved that the association of allergic rhinitis increases 3 times the risk of persistence of childhood asthma [64]. However, the diagnosis of allergic rhinitis may go unnoticed if the parent or caregiver is not actively asked about nasal symptoms [65]. In a study conducted in the United States, for 53% of children with asthma, allergic rhinitis remained undiagnosed until clinically evaluated for the study [66]. It is, therefore, essential to emphasize the importance of appropriate treatment of allergic rhinitis, especially when it is persistent or severe. This helps reduce the need for emergency care and the frequency of asthma attacks, thereby better controlling this comorbidity [62].

As supported by our data, allergic conjunctivitis is a common comorbidity of pediatric asthma. A study showed that among 137 children with conjunctivitis, more than half (56%) also had asthma [67].This implies the inclusion of this important comorbidity in future guidelines on the management of asthma. Likewise, it has already been demonstrated that allergic conjunctivitis is a risk factor for uncontrolled asthma, finding that 52% of patients with allergic conjunctivitis had uncontrolled asthma [68].

Our study examined the impact of rhinitis and allergic conjunctivitis independently, however, several studies have highlighted the impact of the simultaneous presence of two comorbidities on asthma control. For example, there is a combination of allergic rhinitis and atopic dermatitis [26]. In addition, the coexistence of rhinitis and allergic conjunctivitis [27].

This study has certain limitations. First, participants were recruited from a specialist consultation in an MCH, which could introduce selection bias. Children seen in this hospital setting may present with different levels of asthma severity than those seen in primary care settings. A selection bias is also possible, particularly for children in rural areas, who may represent more severe cases due to difficulties in accessing care. Additionally, the data collection period may not have captured seasonal variations in asthma or the effects of fluctuating environmental exposures on disease control. Data collection over a longer period would have been desirable to more comprehensively evaluate these temporal and environmental variations in children's asthma management.

In addition, we suggest that future studies could include strategies such as home visits or samples from multiple care settings, including rural health centers, to obtain a more representative population.

## CONCLUSION

More than half of the children followed in pediatric pneumotherapy consultations at the Marrakech MCH in Morocco suffered from uncontrolled asthma. A set of predictive factors for this uncontrolled asthma were highlighted in the present study. Corrective actions must be applied to ensure good control of childhood asthma, such as 1) educating children and their parents on the prevention of asthma attack triggers, including respiratory infections and exposure to tobacco smoke, and on therapeutic compliance, 2) adequate treatment of comorbidities, particularly rhinitis and allergic conjunctivitis, and 3) strategic actions to make childhood asthma care easily accessible and affordable to all socio-economic classes.

## Figures and Tables

**Table 1:** Sociodemographic characteristics and general information on children and caregivers/parents related to asthma control at the Marrakech University Hospital, Morroco (N = 203).

**Variable**	**Controlled asthma** **(n = 95), n (%)**	**Uncontrolled asthma** **(n = 108), n (%)**	**%** **(N= 203)**	***p*-value**
**Age (in years)** 4-6 7-9 10-11 Mean (6 years, SD) (1,5)	62 (26,26%) 27 (28,42%) 6 (6,31%)	88 (81,48%) 11(10,18%) 9(8,33%)	150 (74%) 38 (18,7%) 15 (7,3%)	0,296
**Gender** Female Male	32(33,68%) 63 (66,31%)	48(44,44Q%) 60(55,55%)	80 (39,4%) 123 (60,6%)	1,117
**Place of residence** Urban Rural	66 (69,47%) 29 (30,52%)	58 (53,70%) 50 (46,29%)	124 (61%) 79 (39%)	0,021*
**Type of accommodation** Apartment Single dwelling Room	76 (80%) 19 (20%) 0	79 (37,14%) 26 (24,07%) 3 (2,77%)	155 (76,4%) 45 (22,2%) 3 (1,4%)	0,724
**Number of persons in household** 3-5 More than 5	55 (57,89%) 40 (42,10%)	56 (51,85%) 52 (48,14%)	54,6% 45,4%	0,131*
**Fathers' level of education** Illiterate Primary Secondary University	3 (3,15%) 49 (51,57%) 21 (22,10%) 22 (23,15%)	26 (24,07%) 28 (25,92%) 29 (26,85%) 25 (23,14%)	29 (14,2%) 77 (38%) 50 (24,6%) 47 (23,2%)	<0,001*
**Mothers' level of education** Illiterate Primary Secondary University	3 (3,15%) 3 (3,15%) 57 (60%) 31 (32,63%)	38 (35,18%) 14 (12,96%) 34 (31,48%) 22 (20,37%)	41 (20,2%) 17 (8,4%) 91 (44,8%) 53 (26%)	<0,001*
**Monthly family income** < 2000 MAD 2000 MAD <I<5000 MAD I> 5000 MAD	33 (34,73%) 50 (52,63%) 12 (12,63%)	50 (46,29%) 52 (48,14%) 6 (5,55%)	83 (41%) 102 (50,2%) 18 (8,8%)	0,095*

(NB. * P -value < 0.05 is considered significant. Variables with P-values ​​less than 0.2 were included in multivariate analysis. SD: standard deviation. MAD: official monetary currency of Morocco.

**Table 2:** Comorbid conditions and trigger factors related to Asthma control among children at the Marrakech University Hospital, Morroco (N = 203).

**Variable**	**Controlled asthma** **(n = 95), n (%)**	**Uncontrolled asthma** **(n = 108), n (%)**	**%** **(N= 203)**	***p*-value**
**Age of asthma** < 2 years >2 years	41 (43,15%) 54 (56,84%)	55 (51%) 53 (49%)	96 (47,29%) 107(52,70%)	0,167
**Past emergency room visits due to asthma** Never 1 time 2 times or more	94 (98,94%) 1 (1,05%) 0 (%)	1 (0,92%) 15 (13,88%) 92(85,18%)	95 (46,79%) 16 (7,88%) 92 (45,32%)	< 0,001*
**Family history of asthma** Yes No	52 (54,73%) 43 (45,26%)	75 (69,44%) 33 (30,55%)	127 (62,56%) 76(37,44%)	0,031*
**Family history of asthma** Mother Father Brothers or sisters Grandparents No family history	14 (14,73%) 15 (15,78%) 7 (7,36%) 16 (16,84%) 43 (45,26%)	24 (22,22%) 26 (24,07%) 13 (12,03%) 12 (11,11%) 33 (30,55%)	38 (18,71%) 41 (20,19) 20 (9,85%) 28 (13,79) 76 (37,43%)	0,082*
**Children's and parents' knowledge of triggers for asthma attacks** Known triggers Unknown triggers	60 (63,15%) 34 (35,78%)	4 (3,70%) 104 (96,29%)	64 (31,52%) 138 (67,98%)	<0,001*
**Asthmatic children's exposure to triggers in the previous month** Exposure to smoking Presence of pets Respiratory tract infection Physical exercise	4 (4,21%) 24 (25,26%) 4 (4,21%) 12 (12,63%)	23 (21,29%) 22 (20,37%) 104 (96,29%) 22 (20,37%)	27(13,30%) 46 (22,66%) 108 (53,20%) 34 (16,74%)	<0,001* 0,406 <0,001* 0,141*
**Comorbidities** Allergic rhinitis Conjunctivitis Eczema GERD No comorbidity	27 (28,42%) 0 (%) 15 (15,78%) 12 (12,63%) 56 (58,94%)	105 (97,22%) 58 (53,70%) 35 (32,40%) 25 (23,14%) 2 (1,85%)	134 (66,00%) 58 (28,57%) 50 (24,63%) 37 (18,22%) 58 (28,57%)	< 0,001* < 0,001* 0,006* 0,053* < 0,001*
**Frequency of asthma follow-up consultations** 1 visit per month 1 every two months 1 every three months	0 31 (32,63%) 64 (67,36%)	1 (%) 41 (%) 66 (%)	1 (0,49%) 72 (35,46%) 130 (64,03%)	0,451
**Therapeutic compliance** Compliance Moderately compliant Non-compliant	65 (68,42%) 22 (23,15%) 8 (8,42%)	12 (11,11%) 15 (13,88%) 81 (75%)	77 (37,93%) 37 (18,22%) 89 (43,84%)	< 0,001*
**Background treatment** Yes No	88 (92,63%) 7 (7,36%)	103 (95,37%) 5 (4,62%)	191 (94,08%) 12 (5,91%)	0,409
**Therapeutic education in the last six months** 1 session or more No session	24 (25,26%) 71 (74,73%)	30 (27,77%) 78 (72,22%)	54 (26,60%) 149 (73,39%)	0,686

*P -value < 0.05 is considered significant. Variables with P-values ​​less than 0.2 were included in the multivariate analysis. GERD: Gastroesophageal reflux desease.

**Table 3:** Multivariable binary regression for predictors of uncontrolled pediatric asthma in CHU, Marrakech, Morocco (N = 203).

**Variable**	**OR**	**(95% CI)**		***p*-value**
		**Lower**	**Upper**	
Place of residence (Urban)	0,51	0,286	0,90	0,022
Fathers' level of education (Illiterate)	7,627	2,027	28,702	0,003
Mothers' level of education (Illiterate)	17,848	4,883	65,242	0,000
Monthly family income (I< 2000 MAD)	3,030	1,035	8,870	0,043
Family history of asthma	1,879	1,057	3,341	0,032
Family history of asthma in mother	2,234	1,004	4,972	0,049
Family history of asthma in father	2,259	1,035	4,931	0,041
Emergency room visits due to asthma (none)	0,003	0,001	0,011	0,000
Knowledge of triggering factors by children or their parents	0,022	0,007	0,064	0,000
Observant to background treatment	0,018	0,007	0,047	0,000
Absence of respiratory tract infection	0,002	0,000	0,007	0,000
The existence of allergic rhinitis	88,148	25,734	301,935	0,000
The existence of conjunctivitis	4,190	1,912	9,185	0,000
Exposure to tobaccosmoke	6,156	2,045	18,534	0,001
Absence of co-morbidities	0,013	0,003	0,056	0,000

CI: Confidence interval. OR: Odds ratio.

## Data Availability

The data and supportive information are available within the article.

## LIST OF ABBREVIATIONS

MCH: = Mother-child hospital
TE: = Therapeutic education
C-ACT: = Childhood Asthma Control Test
PMAQ-3W: = Patient Medication Adherence Questionnaire over the last 3 days and the preceding Weekend
GERD: = Gastro-oesophageal reflux disease
OR: = Odds ratio
CI: = Confidence interval
UHC: = University Hospital Center
GINA: = Global Initiative for Asthma
HCP: = Haut Commissariat au Plan
GORD: = Gastro-Oesophageal Reflux Disease
SPSS: = Statistical Programme for Social Sciences

## STANDARDS OF REPORTING

STROBE guidelines were followed.
